# The Effects of Intravenous Nitroglycerin Bolus Doses in Reducing Hemodynamic Responses to Laryngoscopy and Endotracheal Intubation

**DOI:** 10.1155/2021/6694150

**Published:** 2021-08-03

**Authors:** Pouran Hajian, Shabnaz Sharifi, Mahshid Nikooseresht, Abbas Moradi

**Affiliations:** ^1^Department of Anesthesiology, Besat Hospital, Hamadan University of Medical Sciences, Hamadan, Iran; ^2^Student Research Committee, Hamadan University of Medical Sciences, Hamadan, Iran; ^3^Department of Community Medicine, Hamadan University of Medical Sciences, Hamadan, Iran

## Abstract

**Background:**

Hemodynamic responses to laryngoscopy and endotracheal intubation are transient in most patients. However, in some patients with a history of heart disease, systemic hypertension, or cerebrovascular disease, these may lead to dangerous complications. This study is aimed at determining the effectiveness of intravenous nitroglycerin bolus doses in reducing hemodynamic responses to laryngoscopy and endotracheal intubation. *Material and Method.* In this double-blind randomized controlled trial, 78 patients aged 18 to 65 years were randomly divided into three groups: 1 *μ*g/kg dose of nitroglycerin (first group), 2 *μ*g/kg dose of nitroglycerin (second group), and normal saline or placebo (third group). 26 samples were allocated for each group. Patients' hemodynamic responses to laryngoscopy and endotracheal intubation were measured at different times. Data were analyzed using SPSS V 16.

**Results:**

Patients in the three study groups were similar in terms of age, sex, and weight. There was no significant difference between the mean saturation of peripheral oxygen (SPO_2_) and the mean heart rate between the three groups before endotracheal intubation and 1 to 10 minutes after intubation (*P* > 0.05). The difference of mean arterial blood pressure between study groups was only significant in the first and fifth minutes after intubation. Mean systolic and diastolic blood pressure in the first, third, and fifth minutes after intubation was significantly lower in the intervention groups than the control group (*P* < 0.05). However, no significant difference was observed between the intervention groups. The frequency of systolic blood pressure decrease was significantly different in the first and fifth minutes after intubation in the three study groups (*P* < 0.05).

**Conclusion:**

Bolus doses of 1 and 2 *μ*g/kg nitroglycerin in noncardiac elective surgery prevents the increase of mean systolic, diastolic, and arterial blood pressure but has no significant effect on heart rate after intubation.

## 1. Introduction

Tracheal intubation is usually performed in most patients undergoing surgery and general anesthesia. Laryngoscopy and endotracheal intubation can increase heart rate and blood pressure, make heartbeat irregular, increase catecholamine concentrations, cause myocardial ischemia, and increase intracerebral pressure in susceptible individuals [1].

Hemodynamic responses to laryngoscopy and endotracheal intubation are transient in most patients and may have few consequences. However, in some patients with a history of heart disease, systemic hypertension, or cerebrovascular disease, these may lead to cardiac arrhythmia, left ventricular failure, myocardial ischemia, or cerebral hemorrhage. Proper prescribing, induction of adequate anesthesia, and rapid endotracheal intubation appear to minimize these responses [2, 3].

Various drugs have been used to inhibit or reduce the hemodynamic response, including lidocaine [4], opioids, especially fentanyl [5], and magnesium sulfate [6]. In cases where preadministration with fentanyl is insufficient, nitroglycerin is another option used in some studies to prevent hemodynamic stress-induced ischemia [7]. Nitroglycerin is a vasodilator that acts on vascular smooth muscle and is taken intravenously mixed with 5% dextrose or propylene glycol. Also, it is used as topical or as a spray in the nose. This drug is not toxic and rapidly metabolizes. Its dilating effect on the veins is noticeable, but it also dilates the arteries dose dependently. This drug is used in heart surgeries and hip surgery. Its rapid, reversible effect is similar to nitroprusside and is a reason for its use in anesthesia and CCU [8]. It has a beneficial effect on reducing afterload and preload in patients with heart failure [9].

The present study is aimed at determining the effectiveness of intravenous nitroglycerin bolus doses in reducing hemodynamic responses to laryngoscopy and endotracheal intubation in noncardiac elective surgery.

## 2. Methodology

### 2.1. Study Design and Population

This double-blind randomized controlled trial was performed during 2018-2019 in Besat Hospital in Hamadan. The statistical population included all patients who were candidates for surgery, and the sample consisted of each patient who was a candidate for noncardiac elective surgery. Patients were selected by random methods. The sample size was determined based on the systolic blood pressure and intubation in the study of Singh and Srivani [7], so 78 samples were included.

### 2.2. Inclusion Criteria

The inclusion criteria are as follows:

- Candidate patients for noncardiac
- Elective surgery with ASAI
- Age 18 to 65 years
- Weight less than 100 kg

### 2.3. Exclusion Criteria

The inclusion criteria are as follows:

- Baseline blood pressure less than 50/100 mmHg or higher than 140/90
- Heart rate less than 60 beats per minute
- Conductive disorders of the heart
- More than once attempted piping
- Allergy to anesthetics or any other medication
- Difficulty intubating or prolonging intubation for more than 30 seconds or forcing the patient after intubation
- Use of antihypertensive, antidepressant drugs
- Hypothyroidism and hyperthyroidism, diabetes, and kidney, heart, liver and respiratory diseases

### 2.4. Ethical Considerations

Researchers performed all stages based on the Declaration of Helsinki, and in order to maintain the confidentia**l**ity of information, the data required for the research were collected anonymously. This study was conducted with the permission of the Ethics Committee of Hamadan University of Medical Sciences (IR.UMSHA.REC.1397.297) and was approved by the Iranian Registry of Clinical Trials (IRCT20120215009014N265). Participation in the study was conscious and voluntary. Written consent was obtained from the study participants. Patients were reassured that the study results would be published anonymously.

### 2.5. Data Collection

Data collection tools in this study included two checklists for demographic characteristics and hemodynamic changes. In this double-blind randomized controlled trial, 78 patients were evaluated among the eligible patients. A complete history of illness and medication was taken from all patients during the anesthesia visit. After examination, patients were randomly divided into three equal groups using 6-item randomization blocks: 1 *μ*g/kg dose of nitroglycerin (first group), 2 *μ*g/kg dose of nitroglycerin (second group) during induction, and normal saline or placebo (third group).

Systolic and diastolic blood pressure, mean arterial blood pressure, and oxygen saturation were measured by noninvasive monitoring (saadat Novin1800 Masimo SET) and recorded as baseline. Induction of standard anesthesia was performed in all patients in the same way. Initially, patients were preoxygenated with 6 liters/min of oxygen for 3 minutes. Midazolam 0.01 mg/kg and fentanyl 2 mcg/kg were injected before induction; then, 2.5 mcg/kg propofol and 0.5 mg/kg atracurium were injected. In the intervention and control groups, immediately after induction, nitroglycerin or distilled water, which was poured into a 2 cc syringe of the same shape and color, was injected by the nurse. The intervention group's syringes contained doses of 1 *μ*g/kg and 2 *μ*g/kg of nitroglycerin, and the control group syringes contained 2 cc of distilled water. The syringe label was recorded in the patient checklist. Three minutes after drug injection and at the same time with preoxygenation in patients with Macintosh laryngoscope and portex tube, endotracheal intubation was implanted by an anesthesiologist.

Systolic blood pressure (SBP), diastolic blood pressure (DBP), mean arterial pressure (MAP), heart rate (HR), and SPO_2_ were recorded three minutes after anesthesia induction (just before endotracheal intubation), in 1, 3, 5, and 10 minutes after intubation.

### 2.6. Randomization and Blinding Method

Block randomization was designed to randomize samples in each group. The researcher provided six envelopes of sheet. On the two sheets the letter I 1 means “Intervention 1”, on the other 2 sheets letter I 2 means “Intervention 2” and on the other two sheets the letter C means “Control” were written. An independent researcher s makes random allocation cards (I 1, I 2, and C). The sheets were put in the envelopes and mixed together and placed in a drawer. Another independent researcher referred to each eligible patient, and one envelope randomly was drawn and based on its type; the subjects were assigned to one of the I 1, I 2, or control groups. The patients were not aware of the envelope details. Another independent researcher sees the sheet and informed the physician about which drug should be prescribed for which subject. So, the doctor was not aware of the prescription.

### 2.7. Statistical Analysis

SPSS software version 16 was used to analyze the data. Dispersion tables were used to summarize the data. For comparing mean arterial blood pressure, systolic and diastolic blood pressure, and heart rate, an ANOVA test was used. Also, Chi-square test (Fisher's exact test) was used for comparing the frequency of hypertension, hypotension, bradycardia, and tachycardia. *P* < 0.05 was considered the significance level in this study, and the test power was 80%.

## 3. Results

In this study, 78 patients in three groups were evaluated (26 in each group). Based on the one-way ANOVA test results, the groups were similar in terms of gender (*P* = 0.513), age (*P* = 0.144), and weight (*P* = 0.932) (Table 1).

The mean SPO_2_ before endotracheal intubation and 1 to 10 minutes after intubation did not show a significant difference between the 3 groups (*P* > 0.05).

According to the findings, the mean heart rates before endotracheal intubation and 1 to 10 minutes after intubation were not significantly different in the three groups (*P* > 0.05) (Table 2).

The results of one-way ANOVA and Tukey's post hoc test showed that in the first, third, and fifth minutes the mean systolic and diastolic blood pressure in the placebo group was significantly higher than that in the group receiving 1 *μ*g/kg dose of nitroglycerin and the group receiving 2 *μ*g/kg dose of nitroglycerin (*P* < 0.05) (Table 2).. However, there was no significant difference between the 2 intervention groups (*P* > 0.05) (Table 2).

The results of the one-way ANOVA test and Tukey post hoc test showed that the mean arterial pressure at 1 and 5 minutes after intubation in the placebo group was significantly higher than that in the group receiving 1 *μ*g/kg dose of nitroglycerin (*P* = 0.007) and the group receiving 2 *μ*g/kg dose of nitroglycerin (*P* = 0.004). However, there was no significant difference between the 2 intervention groups (*P* > 0.05) (Table 2).

There was a significant difference between frequency of systolic blood pressure decrease at 1 and 5 minutes after endotracheal intubation between the study groups (*P* < 0.05) (Table 3). Compared to the other groups, the most marked blood pressure decrease regarding the baseline was seen in the group receiving 2 *μ*g/kg dose of nitroglycerin (Table 3).

Considering the frequency of mean arterial blood pressure changes (increase or decrease), there was no significant difference between the groups at different times after intubation (Table 3).

The results showed that there was a significant difference between three study groups in terms of tachycardia and bradycardia in the first, fifth, and tenth minutes after endotracheal intubation (*P* < 0.05) (Table 4).

## 4. Discussion

The present study is aimed at determining the effectiveness of intravenous nitroglycerin bolus doses in reducing hemodynamic responses to laryngoscopy and endotracheal intubation in noncardiac elective surgery. According to the present study's findings, there was no significant difference between the SPO_2_ and the mean heart rate between the 3 groups before endotracheal intubation and 1 to 10 minutes after intubation. In the placebo group, the mean arterial blood pressure (in the first and fifth minutes after intubation) and systolic and diastolic blood pressure (in the 1, 3, and 5 minutes after intubation) were significantly higher than in patients receiving nitroglycerin. However, in terms of hemodynamic data, no significant difference was observed between the intervention groups. The drop in systolic blood pressure was significantly different between groups, but in terms of mean arterial pressure, no significant difference after intubation was observed.

In Singh and Srivani's study, the effectiveness of intravenous nitroglycerin was evaluated against the hemodynamic effects of laryngoscope and intubation in 80 patients aged 20 to 40 years. The intervention group received nitroglycerin at a dose of 2.5 to 5 mcg/min, 5 minutes before intubation. Mean systolic blood pressure in the first and third minutes, diastolic blood pressure in the first, third, and fifth minutes and HR of the first minute in the experimental group were significantly lower than that in the control group [7]. The age range of patients in the present study was 18 to 65 years. Consistent with Singh et al.'s findings, in the present study, systolic, diastolic, and mean arterial blood pressure in patients receiving nitroglycerin in the first and fifth minutes after the intervention were lower than that in the placebo group.

In Pegu et al.'s study, the effects of sublingual nitroglycerin spray plus intravenous fentanyl were compared with nitroglycerin spray alone; 120 patients aged 18 to 60 years in class I and II undergoing elective surgery and general anesthesia were studied in the three groups of 40 people. The first group was the control, the second group received nitroglycerin spray two minutes before intubation, and the third group received nitroglycerin plus fentanyl 5 minutes before intubation. Consistent to the findings of the present study, the results showed that in the first and fifth minutes, the blood pressure in the patients receiving nitroglycerine was significantly lower than that in the control group [10]. However, in our study, the patients were in class I for ASA, and instead of sublingual administration of nitroglycerin, the injectable form was used.

In a double-blind controlled trial of Firoozbakhsh et al., to determine the effectiveness of intravenous nitroglycerin on patients' blood pressure during intubation, 150 patients aged 20 to 50 were investigated. The results showed that the mean systolic blood pressure of patients in the intervention group at 1, 2, and 5 minutes after intubation was significantly lower than that in the control group [11]. Consistent with Firoozbakhsh et al.'s study, in the present study's intervention groups, mean arterial pressure in the first and fifth minutes after intubation and systolic and diastolic blood pressure in the first, third, and fifth minutes were significantly lower than the control group.

In a study by Kumari et al. evaluating the effect of nitroglycerin sublingual spray on reducing the hemodynamic response induced by tracheal intubation, 90 patients aged 18 to 60 were studied. According to the findings, hypertension in the control group and the groups receiving 0.4 and 0.8 mg of nitroglycerin was 60%, 10%, and 10%, respectively. Mean systolic, diastolic, and MAP blood pressure in the control group increased significantly compared to baseline after intubation. In the groups receiving 0.4 and 0.8 mg nitroglycerin, a decreasing trend of systolic, diastolic, and MAP blood pressure was observed one minute after spraying [3]. In terms of sample size, grouping of patients, and age range, the present study's methodology was similar to the study of Kumari et al., with the difference that in the present study, the injectable form was used instead of sublingual nitroglycerin. Consistent with the findings of Kumari et al., in the present study, an increase in blood pressure was observed in the first and third minutes after intubation in the placebo group and a decrease in blood pressure in the first, third, and fifth minutes in the nitroglycerin groups. Contrary to Kumari et al.'s findings, there was no significant difference between the intervention groups (1 and 2 *μ*g/kg nitroglycerin) in terms of mean systolic, diastolic, and MAP blood pressure at different times during the study. This may be due to discrepancies in the route which nitroglycerin was administered.

In a study by Channaiah et al. comparing the effect of intravenous injection of fentanyl and fentanyl plus sublingual nitroglycerin spray on hemodynamic response following endotracheal intubation, the results showed that combined use of fentanyl and nitroglycerin compared with fentanyl alone has no significant difference [12]. In our study, mean of MAP. systolic, and diastolic blood pressure was significantly lower in the groups receiving IV nitroglycerine at 1, 3, and 5 minutes after intubation compared to the placebo group; however, instead of sublingual administration, the IV injection method was used.

## 5. Conclusion

Bolus doses of 1 and 2 *μ*g/kg nitroglycerin in noncardiac elective surgery prevents the increase of mean systolic, diastolic, and mean arterial blood pressure but has no significant effect on the heart rate after intubation.

## Figures and Tables

**Table 1:** Demographic information of the studied subjects.

Variables	Subindex	Placebo	1 *μ*g/kg dose of nitroglycerin	2 *μ*g/kg dose of nitroglycerin	*P* value
Age (year, mean (SD))	—	42.11 (9.2)	40.9 (3.01)	36.13 (8.5)	0.14
Weight (kg, mean (SD))	—	71.9 (36.6)	71.12 (38.32)	70.17 (15.32)	0.93
Sex (frequency, percent)	Male	13 (50)	10 (38.5)	14 (53.8)	0.51
	Female	13 (50)	16 (61.5)	12 (46.2)	

**Table 2:** Comparison of mean (SD) of hemodynamic responses in the studies groups.

Variables	Time of measuring	Placebo	1 *μ*g/kg dose of nitroglycerin	2 *μ*g/kg dose of nitroglycerin	*P* value
Heart rate	Baseline	79.23 (14.08)	90.11 (14.17)	82.84 (13.43)	0.06
	Before endotracheal intubation	83.38 (10.89)	91.30 (20.72)	87.61 (21.25)	0.22
	1 minute after endotracheal intubation	90.50 (14.15)	91.38 (18.97)	100.92 (17.92)	0.058
	3 minutes after endotracheal intubation	89.34 (0.7)	91.00 (14.49)	87.26 (91.94)	0.7
	5 minutes after endotracheal intubation	85.84 (12.11)	83.46 (14.7)	82.11 (16.26)	0.64
	10 minutes after endotracheal intubation	81 (11.01)	77.92 (12.75)	81.38 (13.08)	0.54

Systolic blood pressure	Baseline	124.76 ± 9.25	124.50 ± 11.21	124.96 (8.99)	0.98
	Before endotracheal intubation	105.35 (19.39)	104.08 (11.48)	95.23 (16.12)	0.53
	1 minute after endotracheal intubation	129.92 (25.58)	114.81 (15.46)	110.77 (21.26)	0.004^∗^
	3 minutes after endotracheal intubation	124.27 (18.30)	111.88 (15.91)	109.62 (18.62)	0.008^∗^
	5 minutes after endotracheal intubation	118.62 (14.61)	110.54 (18.55)	105.65 (19.08)	0.03^∗^
	10 minutes after endotracheal intubation	115.68 (19.46)	116.04 (19.030	108.15 (23.74)	0.26

Diastolic blood pressure	Baseline	80.53 ± 7.15	79.30 ± 10.57	81.08 ± 11.40	0.8
	Before endotracheal intubation	66.23 ± 16.33	62.80 ± 10.60	58.11 ± 13.59	0.1
	1 minute after endotracheal intubation	86.69 ± 15.96	89.92 ± 14.76	71.23 ± 20.03	0.001^∗^
	3 minutes after endotracheal intubation	82.76 ± 12.74	70.53 ± 16.71	67.46 ± 15.25	0.001^∗^
	5 minutes after endotracheal intubation	78.19 ± 15.66	67.34 ± 13.95	64.46 ± 15.93	0.004^∗^
	10 minutes after endotracheal intubation	74.96 ± 16.74	70.53 ± 11.58	69.69 ± 13.18	0.39

Mean arterial pressure	Baseline	95.34 ± 10.03	96.03 ± 11.83	94.75 ± 10.71	0.88
	Before endotracheal intubation	79.61 ± 17.42	78.52 ± 10.34	72.11 ± 13.19)	0.12
	1 minute after endotracheal intubation	101.77 ± 19.63	86.38 ± 14.35	85.53 ± 18.9	0.002^∗^
	3 minutes after endotracheal intubation	96.38 ± 13.24	90.46 ± 25.66	83.15 ± 16.40	0.049^∗^
	5 minutes after endotracheal intubation	91.91 ± 13.68	81.92 ± 4.98	78.62 ± 17.81	0.008^∗^
	10 minutes after endotracheal intubation	86.61 ± 11.01	86.46 ± 13.21	84.96 ± 12.34	0.49

**Table 3:** Comparison of the frequency of hemodynamic response changes in the three study groups.

Variables	Placebo	1 *μ*g/kg nitroglycerin	2 *μ*g/kg nitroglycerin	*P* value
1 minute after endotracheal intubation				
Increase of MAP	6 (23.1%)	1 (3.8%)	4 (15.4%)	
Percentage of increase relative to baseline	34.76%	26.51%	24.69%	0.134
3 minutes after endotracheal intubation				
Increase of MAP	1 (3.8%)	2 (7.7%)	2 (7.7%)	
Percentage of increase relative to baseline	33.25%	25.43%	23.71%	0.80
5 minutes after endotracheal intubation				
Increase of MAP	3 (11.5)	2 (7.7%)	1 (3.8%)	
Percentage of increase relative to baseline	33.43%	25.42%	26.73%	0.725
10 minutes after endotracheal intubation				
Increase of MAP	3 (11.5%)	1 (3.8%)	0	
Percentage of increase relative to baseline	32.64%	24.48%	0	0.158
1 minute after endotracheal intubation				
Decrease of MAP	2 (7.7%)	7 (26.9%)	7 (26.9%)	
Percentage of decrease relative to baseline	26.19%	29.40%	35.30%	0.14
3 minutes after endotracheal intubation				
Decrease of MAP	1 (3.8%)	5 19.2%)	8 (30.8%)	
Percentage of decrease relative to baseline	26%	28.53%	30.04%	0.57
5 minutes after endotracheal intubation				
Decrease of MAP	3 (11.5%)	9 (34.6%)	8 (30.8%)	
Percentage of decrease relative to baseline	25.64%	27.43%	32.43%	0.124
10 minutes after endotracheal intubation				
Decrease of MAP	4 (15.4%)	7 (26.9%)	5 (19.2%)	
Percentage of decrease relative to baseline	26%	28.37%	35.41%	0.577

**Table 4:** The frequency of heart rate changes compared to baseline in the study groups.

Variables	Placebo	1 *μ*g/kg nitroglycerin	2 *μ*g/kg nitroglycerin	*P* value
1 minute after endotracheal intubation				
Tachycardia	12 (46.2%)	4 (15.4%)	5 (19.2%)	
Percentage of increase relative to baseline	34.3%	31%	29%	0.028^∗^
3 minutes after endotracheal intubation				
Tachycardia	8 (30.8%)	4 (15.4%)	5 (19.2%)	
Percentage of increase relative to baseline	33%	29%	28.7%	0.284
5 minutes after endotracheal intubation				
Tachycardia	8 (30.8%)	2 (7.7%)	3 (11.5%)	
Percentage of increase relative to baseline	34%	30%	27.53%	
10 minutes after endotracheal intubation				
Tachycardia	6 (23.1%)	2 (7.7%)	0	
Percentage of increase relative to baseline	29.73%	26.30%	0	0.020^∗^
1 minute after endotracheal intubation				
Bradycardia	1 (3.8%)	5 (19.2%)	0	
Percentage of decrease relative to baseline	23%	27%	0	0.023^∗^
3 minutes after endotracheal intubation				
Bradycardia	0	2 (7.7%)	1 (3.8%)	
Percentage of decrease relative to baseline	0	25%	22.36%	0.353
5 minutes after endotracheal intubation				
Bradycardia	0	5 (19.2%)	1 (3.8%)	
Percentage of decrease relative to baseline	0	28.46%	26.94%	0.023^∗^
10 minutes after endotracheal intubation				
Bradycardia	3 (11.5%)	7 (26.9%)	1 (3.8%)	
Percentage of decrease relative to baseline	22%	25.52%	22.34%	0.050^∗^

## Data Availability

All data generated or analyzed during this study are included in this published article.
